# Machine Learning-Based Prediction of Pathological Upgrade From Combined Transperineal Systematic and MRI-Targeted Prostate Biopsy to Final Pathology: A Multicenter Retrospective Study

**DOI:** 10.3389/fonc.2022.785684

**Published:** 2022-04-07

**Authors:** Junlong Zhuang, Yansheng Kan, Yuwen Wang, Alessandro Marquis, Xuefeng Qiu, Marco Oderda, Haifeng Huang, Marco Gatti, Fan Zhang, Paolo Gontero, Linfeng Xu, Giorgio Calleris, Yao Fu, Bing Zhang, Giancarlo Marra, Hongqian Guo

**Affiliations:** ^1^ Department of Urology, Affiliated Drum Tower Hospital, Medical School of Nanjing University, Nanjing, China; ^2^ Institute of Urology, Nanjing University, Nanjing, China; ^3^ Medical School of Southeast University, Nanjing Drum Tower Hospital, Nanjing, China; ^4^ Department of Urology, San Giovanni Battista Hospital, Città della Salute e della Scienza and University of Turin, Turin, Italy; ^5^ Department of Radiology, San Giovanni Battista Hospital, Città della Salute e della Scienza and University of Turin, Turin, Italy; ^6^ Department of Pathology, Affiliated Drum Tower Hospital, Medical School of Nanjing University, Nanjing, China; ^7^ Department of Radiology, Affiliated Drum Tower Hospital, Medical School of Nanjing University, Nanjing, China; ^8^ Department of Urology and Clinical Research Group on Predictive Onco-Urology, APHP, Sorbonne University, Paris, France

**Keywords:** prostate cancer, biopsy, upgrade, prostatectomy, prediction, machine learning

## Abstract

**Objective:**

This study aimed to evaluate the pathological concordance from combined systematic and MRI-targeted prostate biopsy to final pathology and to verify the effectiveness of a machine learning-based model with targeted biopsy (TB) features in predicting pathological upgrade.

**Materials and Methods:**

All patients in this study underwent prostate multiparametric MRI (mpMRI), transperineal systematic plus transperineal targeted prostate biopsy under local anesthesia, and robot-assisted laparoscopic radical prostatectomy (RARP) for prostate cancer (PCa) sequentially from October 2016 to February 2020 in two referral centers. For cores with cancer, grade group (GG) and Gleason score were determined by using the 2014 International Society of Urological Pathology (ISUP) guidelines. Four supervised machine learning methods were employed, including two base classifiers and two ensemble learning-based classifiers. In all classifiers, the training set was 395 of 565 (70%) patients, and the test set was the remaining 170 patients. The prediction performance of each model was evaluated by area under the receiver operating characteristic curve (AUC). The Gini index was used to evaluate the importance of all features and to figure out the most contributed features. A nomogram was established to visually predict the risk of upgrading. Predicted probability was a prevalence rate calculated by a proposed nomogram.

**Results:**

A total of 515 patients were included in our cohort. The combined biopsy had a better concordance of postoperative histopathology than a systematic biopsy (SB) only (48.15% vs. 40.19%, *p* = 0.012). The combined biopsy could significantly reduce the upgrading rate of postoperative pathology, in comparison to SB only (23.30% vs. 39.61%, *p* < 0.0001) or TB only (23.30% vs. 40.19%, *p* < 0.0001). The most common pathological upgrade occurred in ISUP GG1 and GG2, accounting for 53.28% and 20.42%, respectively. All machine learning methods had satisfactory predictive efficacy. The overall accuracy was 0.703, 0.768, 0.794, and 0.761 for logistic regression, random forest, eXtreme Gradient Boosting, and support vector machine, respectively. TB-related features were among the most contributed features of a prediction model for upgrade prediction.

**Conclusion:**

The combined effect of SB plus TB led to a better pathological concordance rate and less upgrading from biopsy to RP. Machine learning models with features of TB to predict PCa GG upgrading have a satisfactory predictive efficacy.

## Introduction

Biopsy-derived tumor grade is currently used for risk stratification and clinical decision-making of prostate cancer (PCa) (1). However, up to 36% of patients with low-grade biopsy upgrade after radical prostatectomy (RP) (2, 3), leading to the potential risk of underestimation and following undertreatment. The risk of the pathological upgrade has been historically predicted using multivariable tools based on clinical parameters (4–6).

In recent years, multiparametric MRI (mpMRI) and mpMRI-targeted biopsy (mpMRI-TB) have been shown to improve the detection of clinically significant PCa (csPCa) (7, 8). Currently, the guideline recommends mpMRI before biopsy and mpMRI-TB combined with 12-core systematic biopsy (SB) for patients with positive mpMRI results (2021). Several models have been developed to predict pathological upgrading using final pathology as the reference (9). A significant added value of mpMRI and mpMRI-TB to the clinical parameters in predicting the risk of upgrading as well as reducing the number of unnecessary repeat prostate biopsies has been previously investigated (9, 10).

Machine learning techniques have been increasingly used in the medical field due to their high accuracy. Compared to the traditional predictive models, machine learning-based models can incorporate a larger number of variables (11). Liu et al. developed a risk model to predict upgrading from biopsy to RP using learning machine-assisted decision-support models (12). However, this tool was developed in patients undergoing transrectal ultrasound (TRUS)-guided SB, with big concerns about its applicability in the era of mpMRI and mpMRI-TB.

Therefore, based on a previous multicenter prospective cohort study of mpMRI-TB (13, 14), we aimed to develop a machine learning-based model for the identification of patients at high risk of upgrading from combined SB and mpMRI-TB to RP.

## Material and Methods

### Study Design

**Figure 1:**
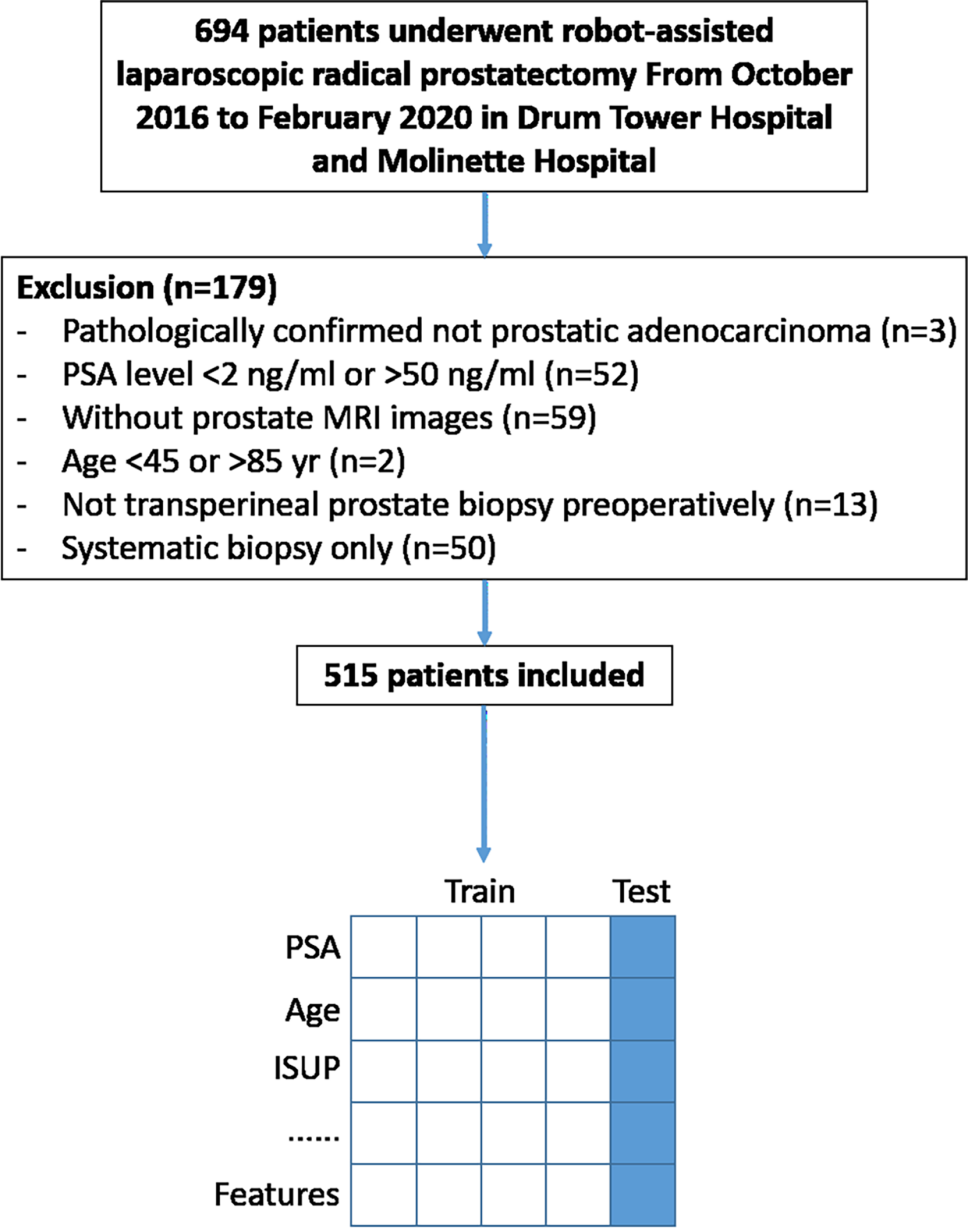
Study flowchart. Data from four out of five included patients were trained for prediction model building and the rest for model validation.

The institutional review board of two hospitals approved this retrospective study from a prospective cohort of mpMRI fusion-targeted biopsies (14) and waived the requirement for informed consent. All patients in this study underwent prostate mpMRI and transperineal systematic plus targeted prostate biopsy under local anesthesia sequentially from October 2016 to February 2020 in Drum Tower Hospital and Molinette Hospital, as previously described (13, 14). Those who were diagnosed with PCa and subsequently treated with robot-assisted laparoscopic RP (RARP) were included. More details about the criteria are shown in **Figure 1**.

### Multiparametric MRI Technique

All patients underwent mpMRI performed with a 1.5- or 3-Tesla system (details shown in **Supplementary Table 1**) (15, 16). Two radiologists with over 10-year experience in urology image analyses supervised the results using the Prostate Imaging-Reporting and Data System (PI-RADS) v2 standards (17). All radiologists had at least 1,000 prostate MRI images of reading experience. The protocol consisted of T2-weighted (T2W) imaging in three planes, diffusion-weighted imaging (DWI) with the calculation of apparent diffusion coefficient (ADC) maps, high b-value images (b > 1,500 s/mm^2^), and dynamic contrast-enhanced imaging (18). In case there was disagreement over the outcome of the image, the radiologists would discuss it until they reached a consensus.

### Biopsy

Biopsy targets were defined as regions of interest (ROIs), which were lesions with PI-RADS score ≥ 3. Before the biopsy, operators annotated targets using an ultrasound system (Esaote Real Time Virtual Sonography, Hitachi Medical Corporation, Tokyo, Japan) with reference to the reports from the two radiologists. All patients underwent transperineal MRI–ultrasonography (MRI-US) fusion biopsy (18G needles with sampling length 17 mm) with a US diagnostic system (MyLab Twice, Esaote S.p.A., Genoa, Italy) consisting of 12-core SB and 2- to 4-core targeted biopsies for each ROI under local anesthesia, as previously described (13, 14).

### Histopathology

Histopathology of prostate biopsies was performed by specialized urological pathologists independent of MRI results. For cores with cancer, grade group (GG) and Gleason score (GS) were determined by using the 2014 International Society of Urological Pathology (ISUP) guidelines (19). csPCa was defined as different criteria according to MRI-FIRST study (csPCa-1, ISUP GG 2 or higher tumors; csPCa-2, ISUP GG 3 or higher tumors) (20).

### Data Collection

Retrospective collection of urology, radiology, and histopathology data for all patients included the following clinical characteristics: histopathology result of biopsy and RP, age, height, weight, body mass index (BMI), pre-biopsy serum prostate-specific antigen (PSA) value (ng/ml), prostate volume, the maximum diameter of lesion, gap days between biopsy and RP, number of cores, and PI-RADS. The length of the prostate on anterior–posterior (AP), head–foot (HF), and right–left (RL) directions were also calculated on T2W mpMRI images.

### Data Visualization and Machine Learning Classifiers

“Upgrade” was defined as ISUP in RP pathology higher than ISUP in biopsy pathology, while “downgrade” was defined as ISUP in RP pathology lower than ISUP in biopsy pathology. These data were visualized under different biopsy situations including SB only, TB only, and SB combined with TB. At the same time, the upgrading and downgrading of different ISUP scores were also visualized. All the visualizations were conducted in Python v3.6.5 (Python Software Foundation) along with the machine learning classifiers with 10-fold cross-validation.

The machine learning algorithms were written using the Python SciKit-learn library except for eXtreme Gradient Boosting (XGBoost), which was written in an individual library called “XgBoost” (21). The logistic regression implemented in SciKit-Learn library was regularized logistic regression. Four classifiers were used including two base classifiers and two ensemble learning-based classifiers. In all classifiers, the training set was 395 of 565 (70%) patients, and the test set was the remaining 170 patients in the two institutions. Among the whole dataset, discrete variables were input to the one-hot encoder, denoting values taken on by categorical (discrete) features. The output would be a sparse matrix where each column corresponded to one possible value of one feature.

Firstly, logistic regression, one of the most classic classifiers to estimate the probability that a patient would have a particular outcome on the basis of related information or clinical characteristics, was used (22). Secondly, a support vector machine (SVM), a mathematical entity for maximizing a particular mathematical function with respect to a given collection of data, was used. The kernel function was used to “space up” the data to higher dimensions, which allowed the data to be linearly separated (23).

A scalable end-to-end tree-boosting system called XGBoost was then used, which could be built with a smaller sample size than most existing systems for ensemble learning-based classifiers. XGBoost is based on gradient tree boosting, an algorithm with which new models are created that predict the residuals of prior models and are then added together for the final prediction (24).

Finally, a random forest within a multiple decision tree model was used (25). Each tree was developed from a bootstrap sample of the training dataset, and each node was the part that was the best among a haphazardly chosen subset of features. The class predictions created by each tree within the forest were amassed, and the ultimate prediction was based on the lion’s share vote (26).

### Parameter Tuning

For all classifiers, we needed to choose optimal parameters based on the training set. A grid search was applied to iterate through each parameter combination. The best parameter set was confirmed by first plotting the receiver operating characteristic (ROC) curve and then selecting the one with the maximum area under the ROC curve (AUC). All classifiers were used to make predictions on the test set.

### Model Evaluation

The performance of these classifiers was mainly evaluated by using overall accuracy, sensitivity, specificity, and AUC. Overall accuracy was the total correct ratio on all test sets. Sensitivity, similar to recall, indicated the proportion of upgraded PCa patients correctly identified by the classifier. Specificity indicated the proportion of patients who were correctly classified as “no-upgrade.” AUC indicated the probability that the classifier would have a higher prediction between “upgrade” and “no-upgrade” cases. All randomizations involved a random seed number of 0.

### Feature Importance

To evaluate the importance of these features and to figure out the most contributed features, the Gini index was leveraged. In the random-forest classifier, every time a node was split on a single feature, the Gini impurity criterion for the two descendent nodes was less than that of the parent node. Adding up the Gini decreases for each feature over all trees in the forest gives fast feature importance, defined as follows:


$$Gini\left(p\right)\;=\;\sum_{k=1}^{K}p_{k}\left(1-p_{k}\right)$$

In the formula, *K* represents the total number of categories and *p_k_* the probability that a case is divided into *k* categories in the case of a single feature (16). Our study was a binary classification, so *K* was equal to 2.

### Nomogram and Evaluation

A nomogram was proposed to visually predict the risk of upgrading. A nomogram was composed of graphical lines of risk factors, points, total points, and upgrading probability. A calibration plot was used to validate how well the nomogram was calibrated. The x-axis of the calibration plot was defined as predicted probability, and the y-axis was defined as the actual probability (27).

Predicted probability was a prevalence rate calculated by a proposed nomogram. Actual probability could be calculated by dividing the number of patients with the same predicted probability by the total number of patients. An ideal line was drawn at a 45° angle in the calibration plot. The nomogram and calibration plot were plotted with R version 3.6.0 in package “rms” version 5.1-4.

## Results

### Baseline Information

From October 2016 to February 2020, a total of 694 patients underwent RARP after prostate biopsy in Drum Tower Hospital and Molinette Hospital. Of these patients, 179 were excluded because they did not meet the criteria. The remaining 515 were included (**Figure 1**). Clinical characteristics of patients are summarized in **Table 1**.

**Table 1:** Clinical characteristics of patients.

Characteristics	N = 515
Age (years)	68.0 (63.0–74.0)
Height (cm)	170.0 (167.0–175.0)
Weight (kg)	72.0 (68.0–77.0)
BMI (kg/m^2^)	24.7 (22.9–27.1)
Pre-biopsy serum PSA value (ng/ml)	8.3 (6.0–12.0)
Anterior-posterior (AP) length (cm)	4.2 (3.9–4.8)
Right–left (RL) length (cm)	4.8 (4.3–5.0)
Head–foot (HF) length (cm)	3.8 (3.4–4.5)
Prostate volume (ml)	35.2 (26.0–48.1)
Maximum diameter of lesion (cm)	1.3 (1.0–1.8)
Number of cores (n)	14.0 (14.0–16.0)
Gap days (d)	18.0 (15.0–25.0)
PI-RADS (n)	
3	85 (16.50%)
4	260 (50.49%)
5	170 (33.01%)

All features except PI-RADS are represented by median (IQR). PI-RADS is represented by n (%).

BMI, body mass index; PI-RADS, Prostate Imaging-Reporting and Data System; PSA, prostate-specific antigen; IQR, interquartile range.

### Upgrading and Downgrading Under Different Biopsy Methods

**Figure 2:**
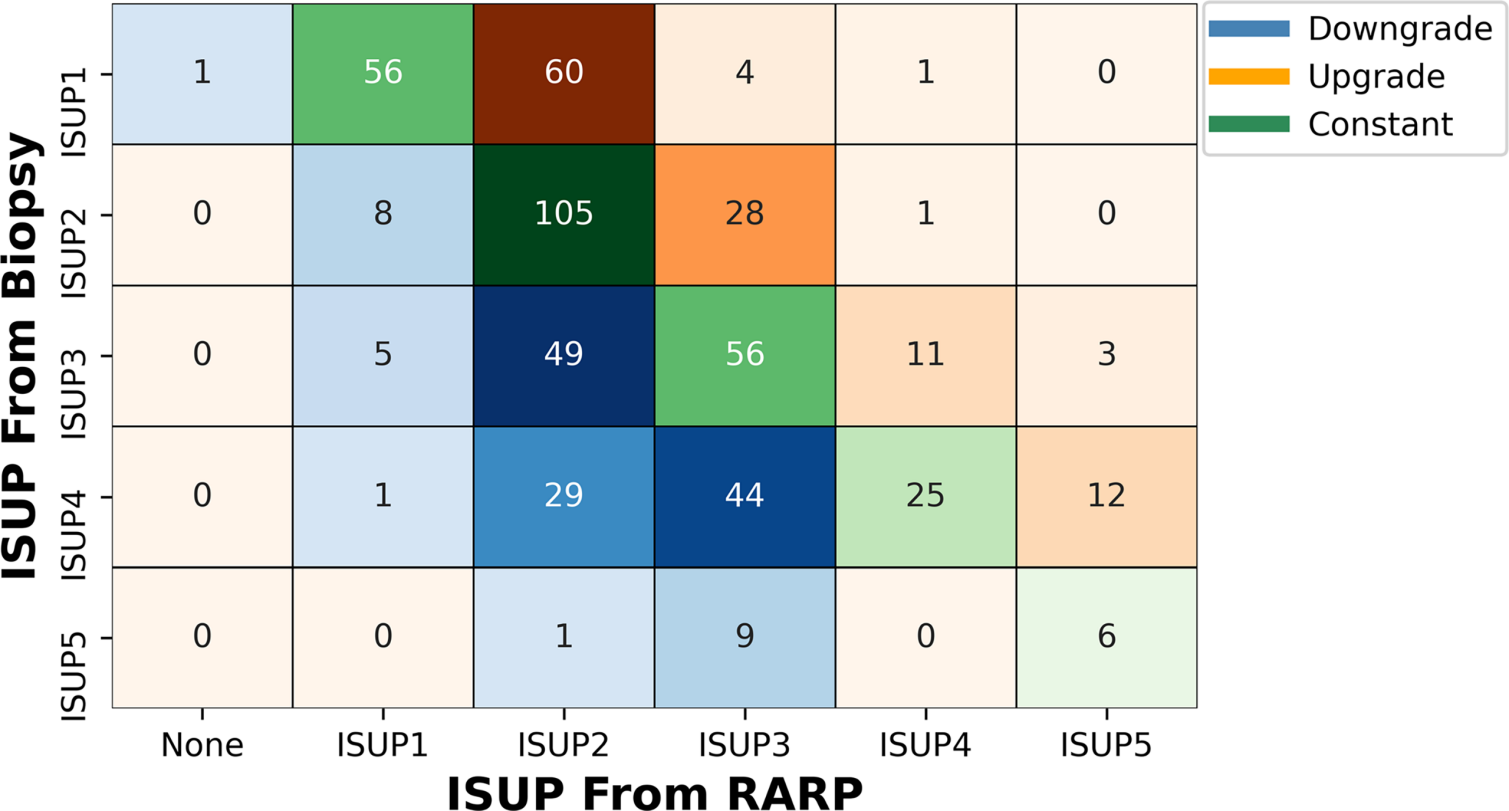
Volume plot of pathological results from combined biopsy to final RARP according to ISUP grade group. The shade of color reflects the number. ISUP, International Society of Urological Pathology; RARP, robot-assisted laparoscopic radical prostatectomy.

Regardless of the combination of SB and TB, of these 515 patients, only 245 (48.15%) cases were in accord with postoperative whole-mount histopathology, accompanied by 120 (23.30%) cases upgrading and 147 (27.18%) cases downgrading (**Figure 2** and **Table 2**).

**Table 2:** Concordance, upgrade, and downgrade of Gleason score according to different biopsy methods.

	Combined biopsy (A)	Systematic biopsy (B)	Targeted biopsy (C)	*p*A vs. B	*p*A vs. C	*p*B vs. C
Concordance	248 (48.15%)	207 (40.19%)	218 (42.3%)	0.012	0.069	0.528
Upgrade	120 (23.3%)	204 (39.61%)	207 (40.19%)	<0.0001	<0.0001	0.899
Downgrade	147 (27.18%)	104 (20.19%)	90 (17.48%)	<0.0001	<0.0001	0.300

The combined biopsy had a better concordance of postoperative histopathology than SB only (48.15% vs. 40.19%, *p* = 0.012). But there was no difference between the combined biopsy and TB only (48.15% vs. 42.33%, *p* = 0.069). The combined biopsy could significantly reduce the upgrading rate of postoperative pathology, in comparison to SB only (23.30% vs. 39.61%, *p* < 0.0001) or TB only (23.30% vs. 40.19%, *p* < 0.0001). Meanwhile, the combined biopsy would lead to a higher postoperative pathology downgrade rate (27.18% vs. 20.19% in SB, *p* = 0.002; and 27.18% vs. 17.48% in TB, *p* < 0.0001) (**Table 2** and **Supplementary Figures 1** **,** **2**). The pathology detail of primary and secondary Gleason patterns from SB and TB to RP is shown in **Supplementary Table 2**.

### Upgrading and Downgrading Under Different International Society of Urological Pathology

Under different ISUP guidelines, 65 (53.28%), 29 (20.42%), 14 (11.29%), and 12 (10.81%) patients with ISUP1, ISUP2, ISUP3, and ISUP4, respectively, had upgrades as compared with postoperative whole-mount histopathology. At the same time, ISUP2, ISUP3, ISUP4, and ISUP5 had 8 (5.63%), 54 (43.54%), 74 (66.67%), and 10 (62.50%) patients with histopathological downgrades, respectively. One patient with confirmed ISUP1 by biopsy was found to have no PCa in the final histopathology (**Figure 2** and **Supplementary Table 3**).

For different levels of upgrading and downgrading, most patients would upgrade to ISUP2 from ISUP1, regardless of the biopsy method. Very few patients upgrade (1.55%) or downgrade (9.13%) two levels or more (**Supplementary Table 3**).

**Figure 3:**
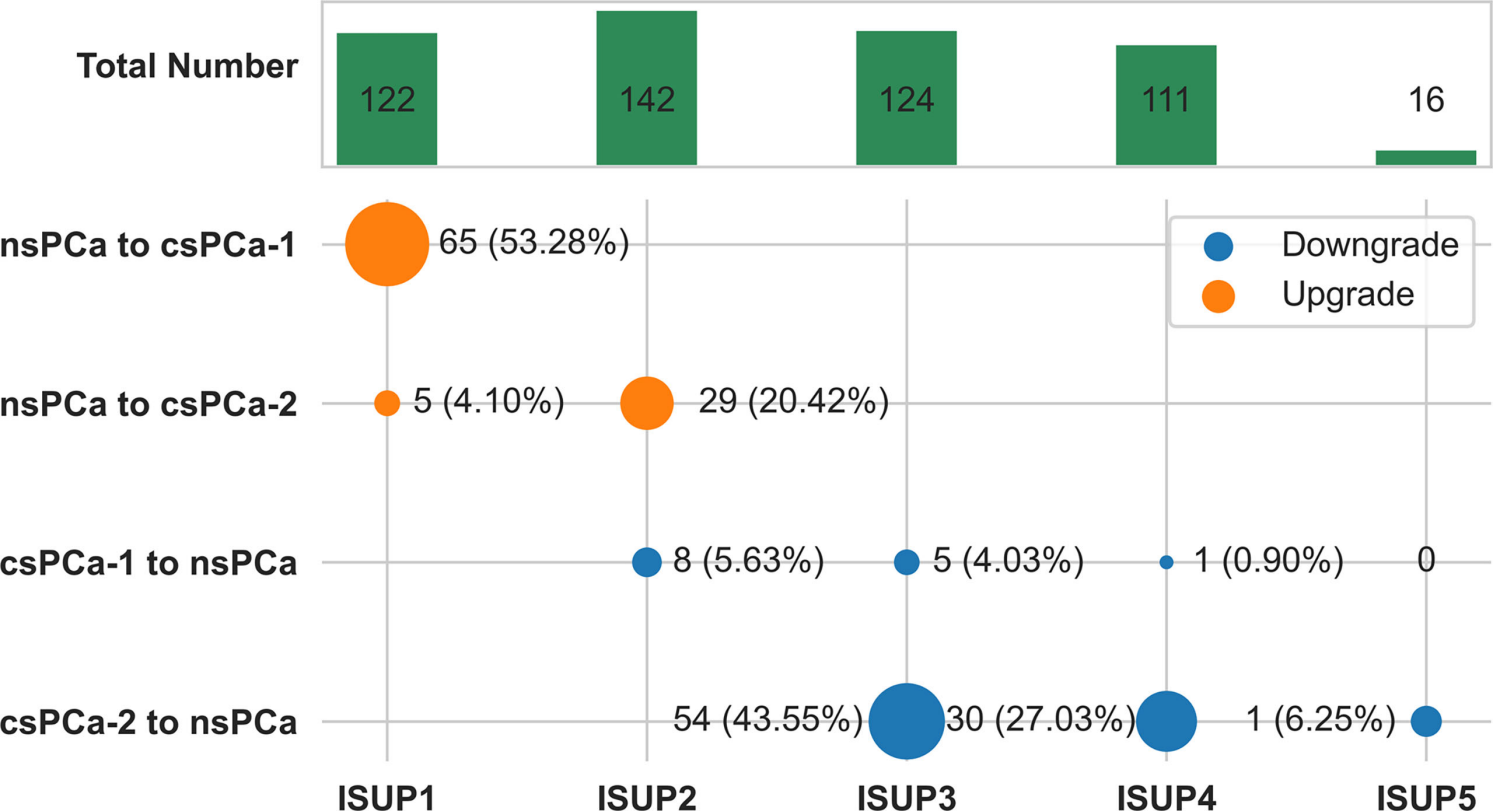
Change plot of clinically significant pathological upgrade or downgrade from combined biopsy to final radical prostatectomy according to ISUP grade group. csPCa-1 was defined as ISUP grade group 2 or higher tumors. csPCa-2 was defined as ISUP grade group 3 or higher tumors. The size of dot reflects the number.csPCa, clinically significant prostate cancer; nsPCa, non-significant prostate cancer; ISUP, International Society of Urological Pathology.

When we identified clinically significant PCa using different ISUP levels (csPC-1: ISUP > 1; csPCa-2: ISUP > 2), there were 65 (53.28%) or 34 (12.88%) cases of clinically significant upgrading and 14 (3.56%) or 85 (33.86%) cases of clinically significant downgrading (**Figure 3**).

### Different Classifier Results

**Figure 4:**
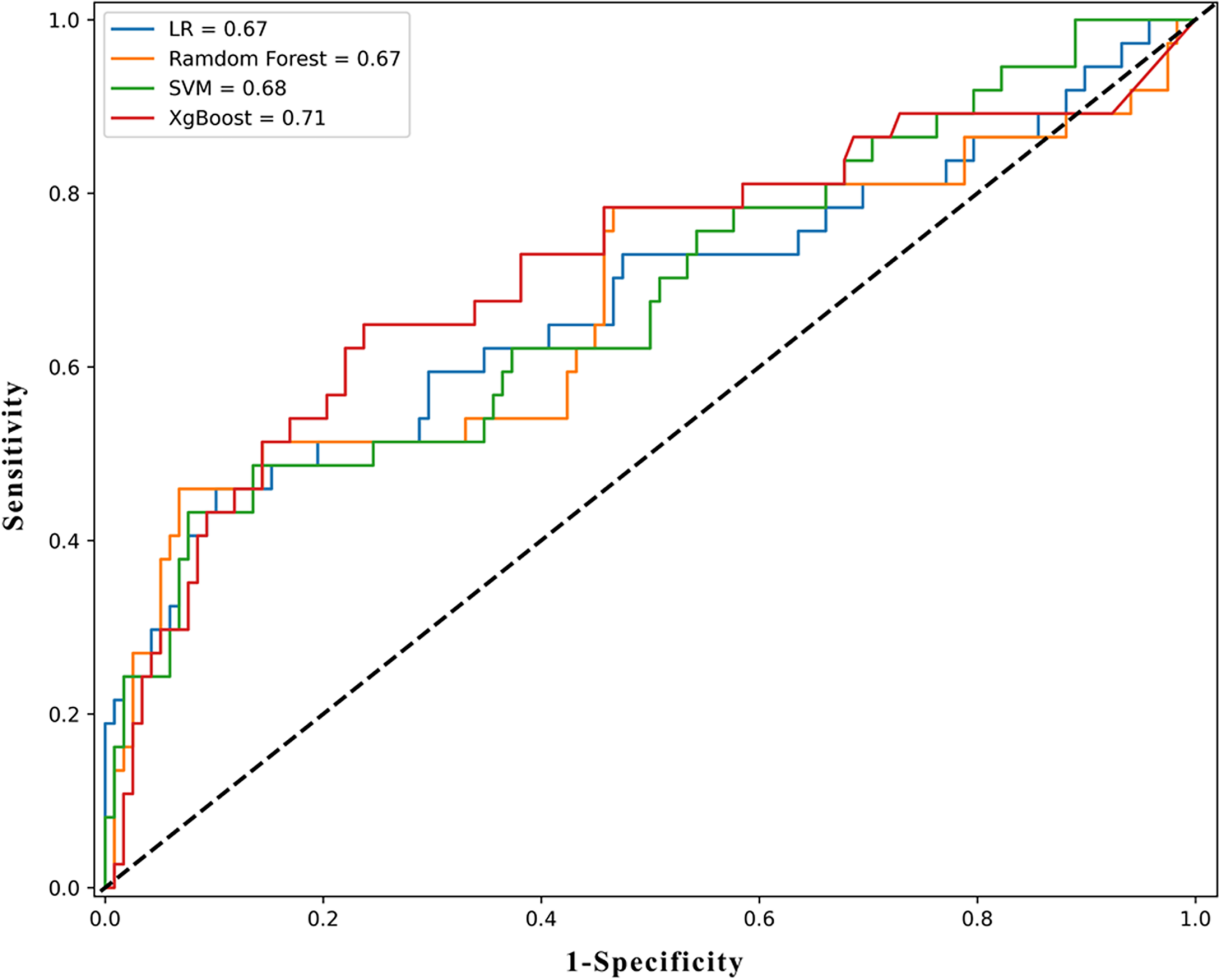
The ROC results of machine learning models. ROC, receiver operating characteristic curve; LR, logistic regression; XGboost, eXtreme Gradient Boosting; SVM, support vector machine.

We used several supervised machine learning algorithms to predict pathology upgrades at RP. We established some dichotomous models in which the label was set to be an upgrade or otherwise. The overall accuracy was 0.703, 0.768, 0.794, and 0.761 for logistic regression, random forest, XGBoost, and SVM, respectively, adjusted for optimal parameters. Their AUC values were 0.674, 0.670, 0.711, and 0.679, respectively. The detailed parameters are described in **Table 3**, and the ROC curve is shown in **Figure 4**.

**Table 3:** Parameters and performance of machine learning algorithms.

Algorithms	Parameters	Overall accuracy	AUC
Logistics regression	C=0.01, penalty=‘l2’	0.703	0.674
Random forest	n_estimators=400, criterion=gini, max_depth=3	0.768	0.670
XGBoost	n_estimators=100, learning_rate=0.01, max_depth=6	0.794	0.711
SVM	C=0.001, kernel=‘linear’, gamma=1*10^-10^	0.761	0.679

XGboost, eXtreme Gradient Boosting; SVM, support vector machine; AUC, area under the receiver operating characteristic curve.

Subsequently, the feature importance calculated using the Gini index was demonstrated, as shown in **Supplementary Figure 3**. The top four important features were ISUP score in a TB, primary Gleason pattern (G1) score in a TB, ISUP score in an SB, and G1 score in an SB. These features were later used in the construction of nomograms.

### Nomogram Construction for Upgrade

**Figure 5:**
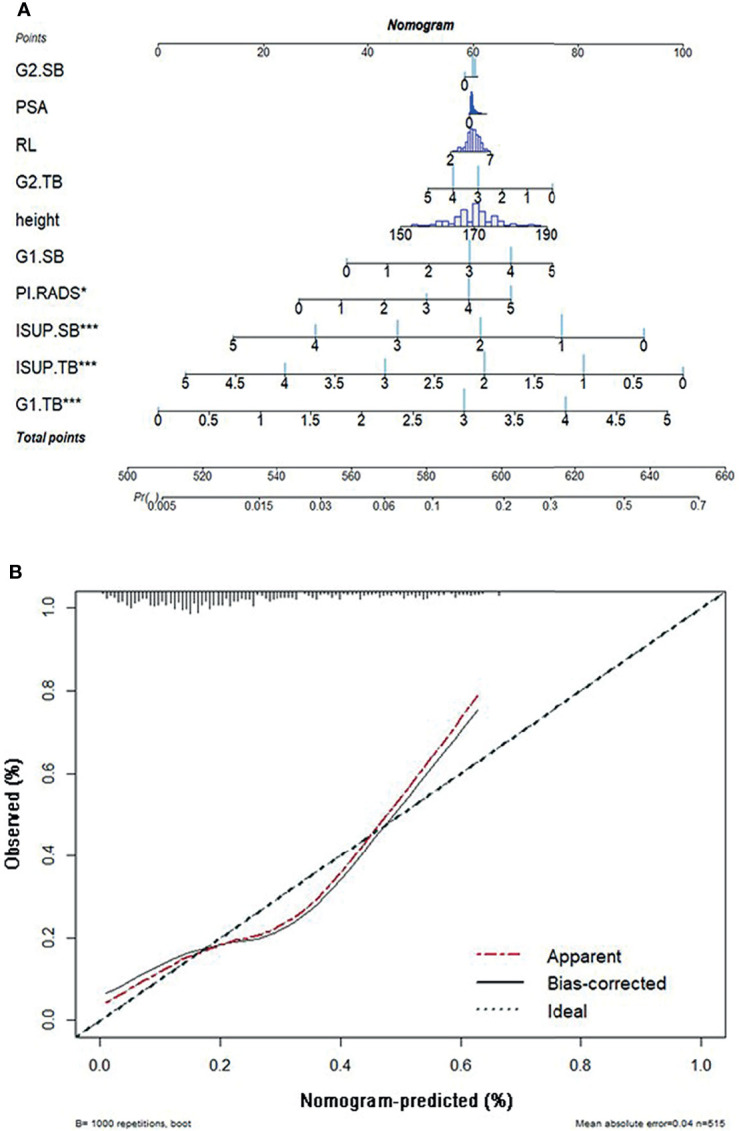
**(A)** The nomogram of pathological upgrade prediction. **(B)** Calibration plots of observed and predicted probability of pathological upgrade. ISUP, International Society of Urological Pathology; TB, targeted biopsy; G1, primary Gleason pattern; SB, systematic biopsy; G2, secondary Gleason pattern; RL, right–left diameter; PSA, prostate-specific antigen; PI-RADS, Prostate Imaging-Reporting and Data System.

We constructed the nomogram to predict the risk of upgrading using 10 risk factors including the top four important features, shown in **Figure 5A**. The longer the line for each feature, the higher the score. When these scores were summed, the higher the total points, the greater the probability of pathology upgrading. When the total points were below 520, the probability of pathology upgrading was less than 10%, while when the total points were above 650, the probability of pathology upgrading would rise to about 0.7.

A calibration plot was created with 1,000 repetitions boot in **Figure 5B**. The mean absolute error was 0.04 in these 515 patients.

## Discussion

Accurate GS is still the strongest decision factor of PCa management and predictor of oncologic outcomes. However, overall pathological characteristics of the prostate cannot be presented by biopsy sampling, as the GG in needle biopsies has poor reproducibility and lack correlation with corresponding RP specimens (28). Approximately 30%–50% of cases will be misled by the biopsy grading system (5, 29). In our multicenter cohort, we found that the overall concordance rate of GG was 48.15% from biopsy to RP.

In recent decades, a relatively growing number of men with PCa are opting for therapies other than RP, such as focal therapy, radiation therapy, or active surveillance, which made biopsy grade more important in therapeutic choices (30). Clinicians are more concerned about the upgrade rates, as the only tissue sampled is from the needle biopsy. The confirmatory biopsy setting was upgraded with a range of 18%–30% of cases, who are previously detected to have PCa and were on active surveillance (31).

Pre-biopsy MRI scan and MRI-guided TB have been widely recommended for men with naïve biopsy or repeat biopsy, as several studies have been proven the improvement of detective rates of PCa or clinically significant PCa (8, 20, 32). Moreover, after RP, pathological upgrade is less likely to occur with MRI-guided TB as previously described (33, 34). Our results showed a significantly lower upgrade rate in the combined biopsy, versus SB or TB alone (both *p* < 0.0001). There was no difference in GG upgrade between SB (39.61%) and TB (40.19%). Some studies indicated that TB alone had better results in pathologic disease upgrade than SB. A possible explanation is in these cohorts of four to six cores, or even saturated biopsy for each lesion was taken (35, 36). Conversely, only two to four cores were performed for each lesion in our protocol.

Similar to other studies (29, 37, 38), the most common pathological upgrade occurred in ISUP GG1 and GG2, accounting for 53.28% and 20.42%, respectively. And these are the two populations of PCa who were potentially selected for active surveillance and/or focal therapy. When we identified csPCa with criteria of ISUP GG3 or higher tumors (csPCa-2 in our study) as previously reported (20), the upgrade rate was 12.88%. But when we made use of other definition criteria, being ISUP GG2 or higher tumors (csPCa-1), the upgrade rate was as high as 53.28%. All these data suggest that upgrading GG may have consequences on clinical outcomes from clinically non-significant PCa to csPCa.

To solve these discrepancies, researchers have sought to predict this pathological upgrade in order to identify patients who are more suitable for active surveillance or needed better and earlier intervention. Several predictive models or analyses of adverse factors were reported, but almost all of them are based on MRI and using PI-RADS (6, 9, 39). Several investigators have included quantitative histologic features to better predict the stage and risk of disease recurrence (40, 41). However, the methods previously used are mainly regression analysis.

Along with the improvement of computer technology, machine learning-based analysis has become better at processing existing data more comprehensively and eliminating more errors (12). Moreover, machine learning methods can construct classifiers with good predictive efficacy. Recently, others combined texture features of mpMRI and machine learning methods to predict GG upgrading (42). They suggested that ADC maps of mpMRI could predict PCa GG upgrading from biopsy to RP non-invasively with satisfactory predictive efficacy. However, their analysis was carried out on a retrospective study of a small group of patients from a single center. Moreover, only TRUS-guided systemic biopsies were included.

To our knowledge, this is the first study to use machine learning to predict pathological GG upgrading including TB. Our transperineal TB cohort was derived from a prospective data collection (13, 14). We used four kinds of supervised machine learning algorithms to predict pathological upgrades from biopsy to RP. Not logistic regression, but XGBoost, was the most accurate algorithm in our cohort. Moreover, we analyzed almost all the relevant characteristics, including TB ISUP score and primary Gleason pattern (G1) score. The top four important features were the ISUP score and G1 score in TB and SB, respectively. We constructed the nomogram to predict the risk of upgrading using 10 risk factors including the top four important features. Similar to another study (6), PI-RADS was one of the most significant predictors, but we demonstrated that the ISUP score and G1 score from TB played a more important role in the prediction model. We hold the opinion that adding features of TB to the prediction model is a notable advance in the selection of candidates for active surveillance. Moreover, the results of the relatively high upgrade rate of ISUP 1 (53.28%) and ISUP2 (20.42%) group might explain the fact that patients with relatively low risk at biopsy suffer from metastasis or even death from PCa. It might suggest that the ISUP 1 and ISUP 2 population, who normally tend to receive active surveillance or focal therapy, might not be suitable candidates.

Some limitations need to be highlighted. Firstly, our study was performed on a retrospective analysis. More prospective data are needed to validate our findings. Secondly, the relatively high number of different operators performed the procedure. Likely, results would have even improved if performed by experienced operators only. In our opinion, the use of multiple operators, together with the multicenter nature, reinforces our results in terms of reproducibility. Despite the limitations, we firmly believe that the principal results of our preliminary study are sufficiently valid.

In conclusion, the combined effect of SB plus TB led to a better pathological concordance rate and the less upgrading rate from biopsy to RP. Machine learning models to predict PCa GG upgrading had satisfactory predictive efficacy. Adding features of TB to the prediction model is a notable advance in the selection of appropriate therapeutic strategies.

## Data Availability Statement

The original contributions presented in the study are included in the article/**Supplementary Material**. Further inquiries can be directed to the corresponding authors.

## Ethics Statement

Written informed consent was obtained from the individual(s) for the publication of any potentially identifiable images or data included in this article.

## Author Contributions

JZ, HG, and GM conceived the study and revised the paper. AM, MO, HH, MG, FZ, LX, YF, and BZ performed the project. YK, YW, GC, and PG collected and analyzed data. JZ, YK, and XQ wrote the draft. JZ, YW, GM, and XQ interpreted the data.

## Funding

This study was supported by grants from the National Natural Science Foundation of China (81602232, 81802535, and 81974394), Natural Science Foundation of Jiangsu Province for Excellent Young Scholars (BK20200051), and Nanjing Medical Science and technique Development Foundation (GRX17127).

## Conflict of Interest

The authors declare that the research was conducted in the absence of any commercial or financial relationships that could be construed as a potential conflict of interest.

## Publisher’s Note

All claims expressed in this article are solely those of the authors and do not necessarily represent those of their affiliated organizations, or those of the publisher, the editors and the reviewers. Any product that may be evaluated in this article, or claim that may be made by its manufacturer, is not guaranteed or endorsed by the publisher.
