# Supplementary figures and images for: Machine Learning-Based Prediction of Pathological Upgrade From Combined Transperineal Systematic and MRI-Targeted Prostate Biopsy to Final Pathology: A Multicenter Retrospective Study
Source: Front Oncol. 2022 Apr 7;12:785684. doi: 10.3389/fonc.2022.785684 (PMC9021959; doi:10.3389/fonc.2022.785684)

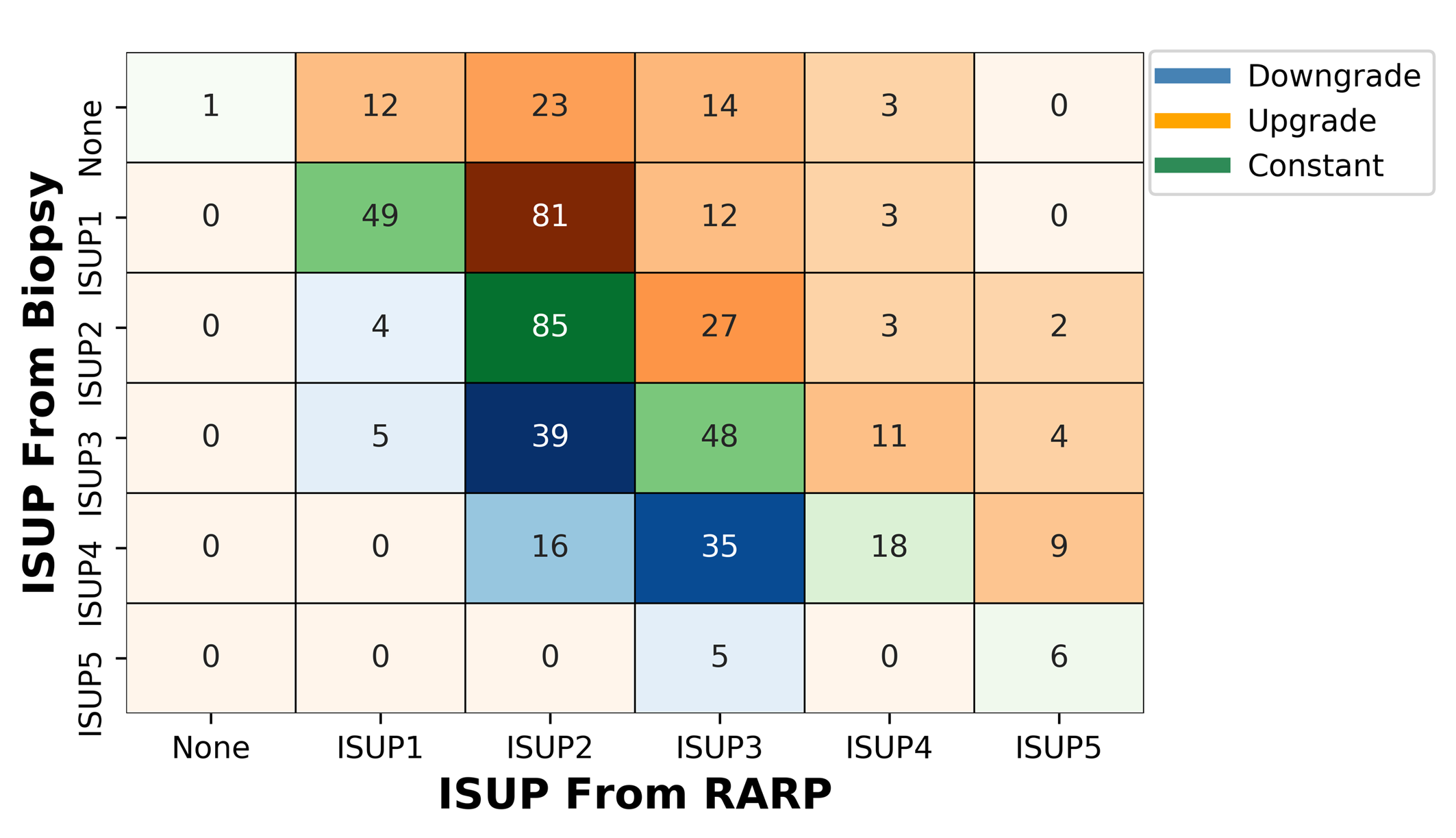

Supplement: Supplementary file 1 [file Image_1.tif]

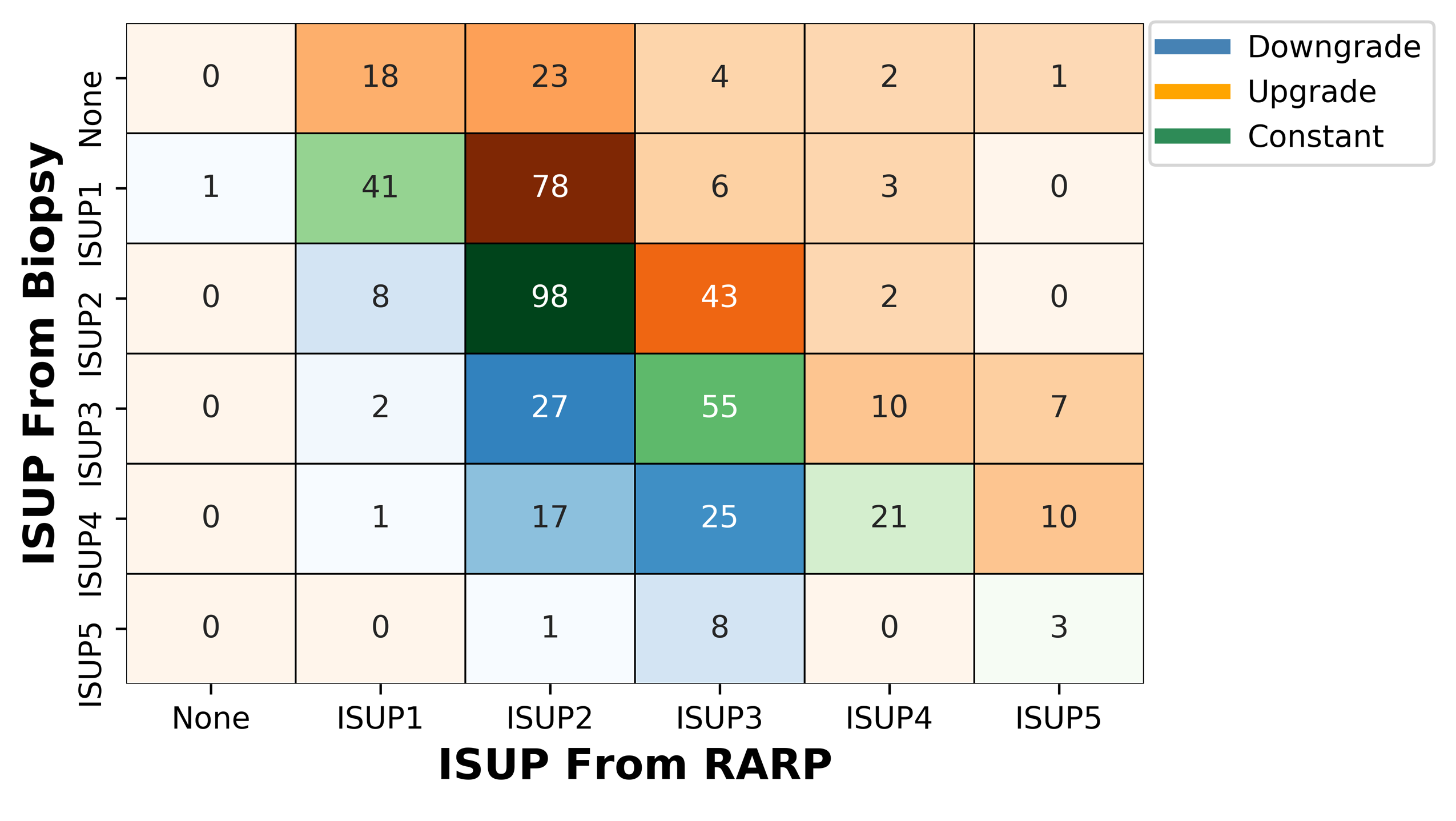

Supplement: Supplementary file 2 [file Image_2.tif]

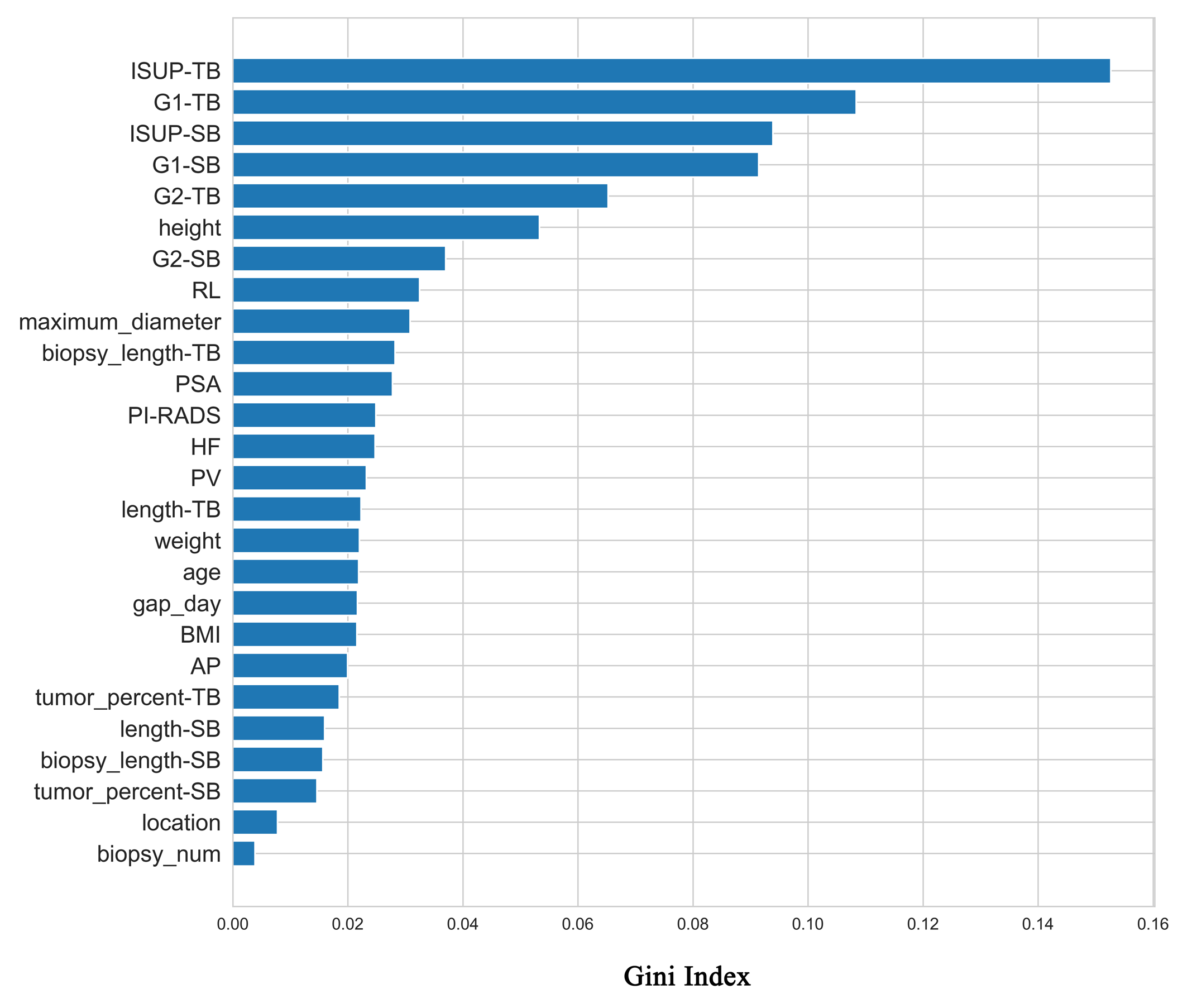

Supplement: Supplementary file 3 [file Image_3.tif]
