# Supplementary material for: Machine Learning-Based Prediction of Pathological Upgrade From Combined Transperineal Systematic and MRI-Targeted Prostate Biopsy to Final Pathology: A Multicenter Retrospective Study
Source: Front Oncol. 2022 Apr 7;12:785684. doi: 10.3389/fonc.2022.785684 (PMC9021959; doi:10.3389/fonc.2022.785684)
Supplement: Supplementary file 4 [file Table_1.doc]

**Supplementary Tables**

**Supplementary Table 1.** Multiparametric MRI acquisition

| 3.0-TMR scanner (Achieva 3.0T TX dual-source parallel RF excitation and transmission technology, Philips Medical Systems, The Netherlands) |
| --- |
| 32-channel phased array coil |
| Transverse/coronal/sagittal (18 slices, thickness3 mm/gap 0.5 mm, TR 3744 ms, TE 120 ms, number of signals acquired 2, resolution 1.49 mm × 1.51 mm) T2-weighted turbo spin-echo (TSE) images |
| Diffusion-weighted imaging, spin-echo–echo-planar images (18 slices, thickness 6 mm, intersection gap 1 mm, TR 925/TE 41 ms, number of signals acquired 1, resolution 3 mm × 3 mm, b-factor 0/800 s/mm2) |
| Thigh-resolution isotropic volume with fat suppression after gadolinium injection (133 slices, thickness 3 mm, no intersection gap, TR 3.1/TE 1.46 ms, number of signals acquired 1, resolution 1.49 mm × 1.51 mm, dynamic scan time 00:06.9) |
| Mappings of the apparent diffusion coeffcient (ADC) from b 0 and b 1000 images of DWI using the Philips WorkStation software (Extended Workspace, EWS) |

**Supplementary Table 2. Postoperative pathology results of patients compared to biopsy pathology of primary and secondary Gleason patterns**

| Results , n (%) | N = 515 |
| --- | --- |
| Systematic Biopsy G1 |  |
| 0 | 53 (10.29%) |
| 3 | 268 (52.04%) |
| 4 | 190 (36.89%) |
| 5 | 4 (0.78%) |
| Systematic Biopsy G2 |  |
| 0 | 53 (10.29%) |
| 3 | 253 (49.13%) |
| 4 | 198 (38.45%) |
| 5 | 11 (2.14%) |
| Systematic Biopsy ISUP |  |
| 0 | 53 (10.29%) |
| 1 | 145 (28.16%) |
| 2 | 121 (23.50%) |
| 3 | 107 (20.78%) |
| 4 | 78 (15.14%) |
| 5 | 11 (2.14%) |
| Targeted Biopsy G1 |  |
| 0 | 48 (9.32%) |
| 3 | 281 (54.56%) |
| 4 | 183 (35.53%) |
| 5 | 3 (0.58%) |
| Targeted Biopsy G2 |  |
| 0 | 48 (9.32%) |
| 3 | 231 (44.85%) |
| 4 | 225 (43.69%) |
| 5 | 11 (2.14%) |
| Targeted Biopsy ISUP |  |
| 0 | 48 (9.32%) |
| 1 | 129 (25.05%) |
| 2 | 151 (29.32%) |
| 3 | 101 (19.61%) |
| 4 | 74 (14.37%) |
| 5 | 12 (2.33%) |

ISUP, International Society of Urologic Pathology; G1, primary Gleason pattern; G2, secondary Gleason pattern.

**Supplementary Table 3.** Upgrade and downgrade under different ISUP

|  | None | ISUP1 | ISUP2 | ISUP3 | ISUP4 | ISUP5 | Total |
| --- | --- | --- | --- | --- | --- | --- | --- |
| RARP | 1 | 70 | 244 | 141 (100%) | 38 (100%) | 21 (100%) | 515 |
| Combined biopsy | - | 122 | 142 | 124 | 111 | 16 | 515 |
| Upgrade | - | 65 (53.28%) | 29 (20.42%) | 14 (11.29%) | 12 (10.81%) | - | 120 (23.3%) |
| Downgrade | - | 1  (0.82%) | 8  (5.63%) | 56 (43.54%) | 74 (66.67%) | 10 (62.5%) | 147 (27.18%) |
| upgrade  ∆Grade > 1 | - | 5  (4.10%) | 1  (0.70%) | 3  (2.42%) | - | - | 9  (1.55%) |
| downgrade  ∆Grade > 1 | - |  |  | 7  (5.65%) | 30  (27.03%) | 10  (62.50%) | 47  (9.13%) |

ISUP, International Society of Urologic Pathology; RARP, robot-assisted laparoscopic radical prostatectomy; None, prostate cancer was confirmed in biopsy, but not found on specimen of radical prostatectomy.
